# The short isoform of MS4A7 is a novel player in glioblastoma microenvironment, M2 macrophage polarization, and tumor progression

**DOI:** 10.1186/s12974-023-02766-1

**Published:** 2023-03-21

**Authors:** Bowen Ni, Guanglong Huang, Runwei Yang, Ziyu Wang, Haimin Song, Kaishu Li, Yunxiao Zhang, Kezhi Wu, Guangwei Shi, Xiran Wang, Jie Shen, Yawei Liu

**Affiliations:** 1 Department of Neurosurgery & Medical Research Center, Shunde Hospital, Southern Medical University (The First People’s Hospital of Shunde Foshan), 1# Jiazi Road, Foshan, 528300 Guangdong China; 2 Department of Neurosurgery, Nanfang Hospital, Southern Medical University, Guangzhou, Guangdong China; 3 Department of Neurosurgery, Ganzhou People’s Hospital, Ganzhou, Jiangxi China; 4 Department of Neurosurgery, The Sixth Affiliated Hospital of Guangzhou Medical University, Qingyuan, Guangdong China; 5 Department of Endocrinology and Metabolism, Shunde Hospital, Southern Medical University (The First People’s Hospital of Shunde Foshan), 1# Jiazi Road, Foshan, 528300 Guangdong China

**Keywords:** MS4A7, Protein isoform, Alternative splicing, Glioblastoma, Glioma-associated macrophages

## Abstract

**Background:**

The unique intracranial tumor microenvironment (TME) contributes to the immunotherapy failure for glioblastoma (GBM), thus new functional protein targets are urgently needed. Alternative splicing is a widespread regulatory mechanism by which individual gene can express variant proteins with distinct functions. Moreover, proteins located in the cell plasma membrane facilitate targeted therapies. This study sought to obtain functional membrane protein isoforms from GBM TME.

**Methods:**

With combined single-cell RNA-seq and bulk RNA-seq analyses, novel candidate membrane proteins generated by prognostic splicing events were screened within GBM TME. The short isoform of MS4A7 (MS4A7-s) was selected for evaluation by RT-PCR and western blotting in clinical specimens. Its clinical relevance was evaluated in a GBM patient cohort. The function of MS4A7-s was identified by in vitro and in vivo experiments. MS4A7-s overexpression introduced transcriptome changes were analyzed to explore the potential molecular mechanism.

**Results:**

The main expression product, isoform MS4A7-s, generated by exon skipping, is an M2-specific plasma membrane protein playing a pro-oncogenic role in GBM TME. Higher expression of MS4A7-s correlates with poor prognosis in a GBM cohort. In vitro cell co-culture experiments, intracranial co-injection tumorigenesis assay, and RNA-seq suggest MS4A7-s promotes activation of glioma-associated macrophages’ (GAMs) PI3K/AKT/GSK3β pathway, leading to M2 polarization, and drives malignant progression of GBM.

**Conclusions:**

MS4A7-s, a novel splicing isoform of MS4A7 located on the surface of GAMs in GBM TME, is a predictor of patient outcome, which contributes to M2 polarization and the malignant phenotype of GBM. Targeting MS4A7-s may constitute a promising treatment for GBM.

**Supplementary Information:**

The online version contains supplementary material available at 10.1186/s12974-023-02766-1.

## Background

Glioblastoma (GBM) is the most prevalent primary malignancy in the adult central nervous system, with a median overall survival (OS) of only 14.6 months [1, 2]. Little important progress has been made in the management of GBM patients in recent decades [3, 4]. The current main treatment for GBM is maximum feasible surgical resection followed by radiotherapy and temozolomide (TMZ) chemotherapy [5]. Although GBM is prone to drug resistance, several studies have explored ways to sensitize TMZ efficacy in recent years [6]. Importantly, the potential of immunotherapy against GBM is highly attractive, as it has achieved great success in the treatment of various tumors [7]. However, there are almost no popular targets for immunotherapy which are virtually applicable to GBM, largely because of the GBM’s unique tumor microenvironment (TME), which is quite distinct from other tumors’ [8–10]. Therefore, the search for novel functional targets specific to GBM TME seems to be a key issue to be addressed [11–13].

Substantial efforts have been devoted to the GBM microenvironment [14, 15]. Future research demands more specific targets, and protein diversity should be emphasized [16]. Alternative splicing (AS) is a prevalent mechanism in complex organisms by which multiple protein isoforms can be produced from a single gene, resulting in protein diversity [17–19]. Some of these isoforms have distinct or even opposite functions [20]. It is of great importance that recent studies including ours, have shown that AS plays an important role in promoting the malignant phenotype of GBM [21–24].

To develop a kind of immunotherapy that is at ease of intervention, functional cell surface proteins would be ideal [25]. Accordingly, in this study, we used a combination of single-cell RNA-sequencing (scRNA-seq), TCGA bulk RNA-sequencing (RNA-seq) data, and prognostic splicing events analyzed from SpliceSeq database to identify specific protein isoforms located in plasma membrane of cells in the GBM TME and to characterize their function [26].

The present study uncovered MS4A7-s, a short isoform of MS4A7, as a key regulator in glioma-associated macrophages (GAMs), which promotes the M2 polarization and predicts poor clinical outcome.

## Materials and methods

### Clinical specimens

All glioma samples were obtained after surgical resection from patients admitted to the Department of Neurosurgery, Nanfang Hospital (NFH), Southern Medical University, China, and the corresponding clinical data were collected. The glioma specimens were obtained for pathological examination and cell isolation. This study was approved by the Ethics Committee of Nanfang Hospital, Southern Medical University. Written informed consent was provided by all patients. Clinical data of patients were obtained from Nanfang Hospital (Additional file 2).

### Cell culture

Established human GBM cell line U87MG was purchased from the American Type Culture Collection (ATCC, USA). Established human microglia cell line HMC3 was a kind gift from Chaofu Mao of Nanfang Hospital. Cell lines were cultured with DMEM (Thermo Fisher, 11,320,033, USA) supplemented with 10% fetal bovine serum (Biological Industries, 040011ACS, Israel) and penicillin–streptomycin (100 U/mL) (Gibco, 15,140,122, USA) in humidified incubators at 37 °C with 5% CO_2_.

### Lentivirus for infection in HMC3 cells

For lentiviral gene overexpression vectors, nucleotide sequences coding MS4A7-l and MS4A7-s were constructed (NCBI Reference Sequence, NM_021201.5 and NM_206938.2) with titers of 3.88 × 10^8^ TU/mL and 4.00 × 10^8^ TU/mL, respectively, which were ordered from the OBiO Technology (Shanghai, China).

### Reverse transcription polymerase chain reaction (RT-PCR)

Total RNA was extracted from HMC3 cells or clinical tissues by using RNA extraction kits (FOREGENE, RE-03111, China), followed by reverse-transcript PCR (RT-PCR) by using the PrimeScript RT master mix (TaKaRa, RR047A, Japanese), according to the manufacturer’s protocols. Subsequently, the resulting complementary DNA (cDNA) was used as templates for PCR amplification by using the 2 × ChamQ SYBR qPCR Master Mix (Vazyme, Q311-02-AA, China) and 2 × Accurate Master Mix (Accurate Biology, A0211, China) in a CFX96 Real-Time PCR System (Bio-Rad). For qRT-PCR, the standard ΔΔCT method was used to calculate the expression levels of targeted messenger RNAs (mRNAs), using GAPDH as a reference gene. Primers for targeted mRNAs are listed in Additional file 3. For PCR, the PCR product was electrophoresed on agarose gels. Then the quantification was performed using ImageJ software (version 1.53e) based on the grayscale values of the bands in the electrophoresis images.

### Protein extraction and western blotting

Protein extraction and western blotting were performed as previously described [24].

### Antibodies used

Rabbit anti-MS4A7 antibody (Abmart, China, #PH5636S), Rabbit anti-AKT antibody (Proteintech, China, #10,176–2-AP), Rabbit anti-GSK3β antibody (Proteintech, China, #22,104–1-AP), Rabbit anti-Phospho-GSK3β(Ser389) antibody (Proteintech, China, #14,850–1-AP), Rabbit anti-Phospho-AKT (Ser473) antibody (CST, China, #4060), Rabbit anti-GAPDH antibody (Proteintech, China, # 60,004–1-Ig).

### Cell proliferation assay

The proliferation ability of HMC3 cells or U87MG cells co-cultured with conditioned medium of HMC3 cells was monitored using a CCK8 kit (GlpBio, USA, #GK10001). For CCK8 assay of U87 cells, the conditioned medium from control, MS4A7-s OE and MS4A7-l OE HMC3 cells was collected, mixed with fresh medium in a 1:2 ratio and used for culturing different groups of U87 cells spread in 96 wells, respectively. CCK8 reagent was added at 72 h and the OD at wavelength at 450 nm was measured after 1 h of incubation. A 5-ethynyl-2′-deoxyuridine (kFluor555-Edu) staining assay, an EdU assay kit (Nanjing KeyGen Biotech Co., Ltd. #KGA331-100 and #KGA337-100) was used to assess U87MG cell proliferation, after co-cultured with HMC3 cells for 48 h. The EdU incorporation assay was carried out using EdU detection kits according to the manufacturer’s instructions.

### Animal experiment

Twenty-one male BALB/C nude mice (4-week-old, weighing 20 g) were obtained from SPF (Beijing) biotechnology co., LTD. The animals were maintained in accordance with the Association for Assessment and Accreditation of Laboratory Animal Care criteria, and all studies were approved by the Institutional Animal Care Committee. For the injection of cells, the mice were fixed in the brain localizer, and the skin was cut along the middle of the mouse head with a scalpel to find the fontanelle, 2 mm to the right and 3 mm posterior, and holes were punched in the skull with an electric drill. The cell suspension was then injected with a micro-syringe. Intracranial co-injection of GBM cells with HMC3 cells was performed at a ratio of 4:3. Finally, 7 nude mice in each group completed the operation and survived to 2 weeks after the operation. MRI was performed using a small animal scanner (PharmaScan 70/16, USA). The mice were carefully monitored and euthanized when symptoms such as lethargy and cachexia developed.

### Phagocytosis assay

HMC3 cells (2 × 10^4^) were cultured in 24-well plate. To prepare a Latex Beads–Rabbit IgG–FITC solution, beads solution were diluted 1:1000 according to manufacturer’s protocol. The original medium was replaced with Latex Beads–Rabbit IgG–FITC solution and incubated for 8 h.

### Sample preparation and 10 × Genomics scRNA-seq

GBM samples were maintained on ice and sent to the laboratory within 1 h of surgical isolation. GBM samples were washed twice with pre-cooled Hank’s balanced salt solution (HBSS, Thermo Fisher, 88,284, USA), during which apparent vessels and pia matter were removed. Samples were minced with a scalpel and then enzymatically digested with a Tumor Dissociation Kit (Miltenyi Biotec, 130–095-929, Germany) on a gentleMACS Octo Dissociator with heaters (Miltenyi Biotec, 130–096-427, Germany) according to the manufacturer’s instructions. Subsequently, cells from GBM tissue samples were resuspended in DMEM (Thermo Fisher, 11,320,033, USA) containing 10% fetal bovine serum (FBS) (Thermo Fisher, 10,099,141, USA) and filtered through a 70-μm nylon filter (Corning Falcon, 431,751, USA). The filtered cells were subjected to 1 × Red Blood Cell Removal Solution (Biogems, 64,010–00-100, USA) for 5 min to remove red blood cells, and then the cell debris was removed with Debris Removal Solution (Miltenyi, 130–109-398, Germany). Following two washes and resuspension in PBS solution containing 0.4% bull serum albumin (BSA) (Thermo Fisher, AM2616, USA), the cells were mixed with Trypan Blue (Thermo Fisher, T10282, USA) to assess their viability. Then, the appropriate volume for each sample was calculated for a target capture of 6,000 cells according to the user guide of the Single Cell 3’ Reagent Kit v2 (10 × Genomics company, 120,237–16, USA). Single-cell droplet generation, reverse transcription, and cDNA library preparation were performed according to the manufacturer’s protocols. Finally, the libraries were sequenced on an Illumina NovaSeq 6000 with 150 bp paired-end sequencing.

### scRNA-seq data processing and the identification of cells

The CellRanger 2.2.0 (10 × Genomics) analysis pipeline was used to generate a digital gene expression matrix from these data according to its guidelines. The raw digital gene expression matrix (UMI counts per gene per cell) was filtered, normalized, and clustered using R 3.5.2 software. Cell and gene filtering was performed as follows. For each detected cell, UMIs were less than the (1-doublet rate) to exclude multiplets. The multiplet rates were determined according to the user guide provided by 10 × Genomics. The UMI was larger than the 8th percentile to exclude ambient bias. Mitochondrial RNA was less than the 90th percentile to exclude devitalized cells. scRNA-seq analysis of all our samples were performed by using Seurat and ClusterProfiler packages, following the reference manual. Cell type-specific markers and GO enrichment results were combined to classify the identity of each cluster. Gene expression profiling and correlation analysis of MS4A7 in different kinds of tumors in TCGA database were carried out by using Gene Expression Profiling Interactive Analysis (GEPIA2: http://gepia2.cancer-pku.cn/#index) [27].

### RNA-sequencing (RNA-seq) data analysis

Total cellular RNA was extracted from HMC3 cells and then sent to Novogene (Beijing, China) for RNA-seq.

### Statistics

The statistical analyses were performed in GraphPad (8.0). Survival analysis of the data from the TCGA GBM cohort was performed in the GEPIA2 platform or R software, using the Kaplan–Meier method to calculate the survival curves (71). Significance cut-off: ns, not significant; *P < 0.05; **P < 0.01; ***P < 0.001; and ****P < 0.0001.

### Data availability

The scRNA-seq data used in this study are available at the Genome Sequence Archive (https://ngdc.cncb.ac.cn/gsa/) with the accession number of CRA002498.

## Results

### MS4A7, significantly elevated in GBM tissue, is selectively expressed in GAMs and closely related to M2 polarization

**Fig. 1:**
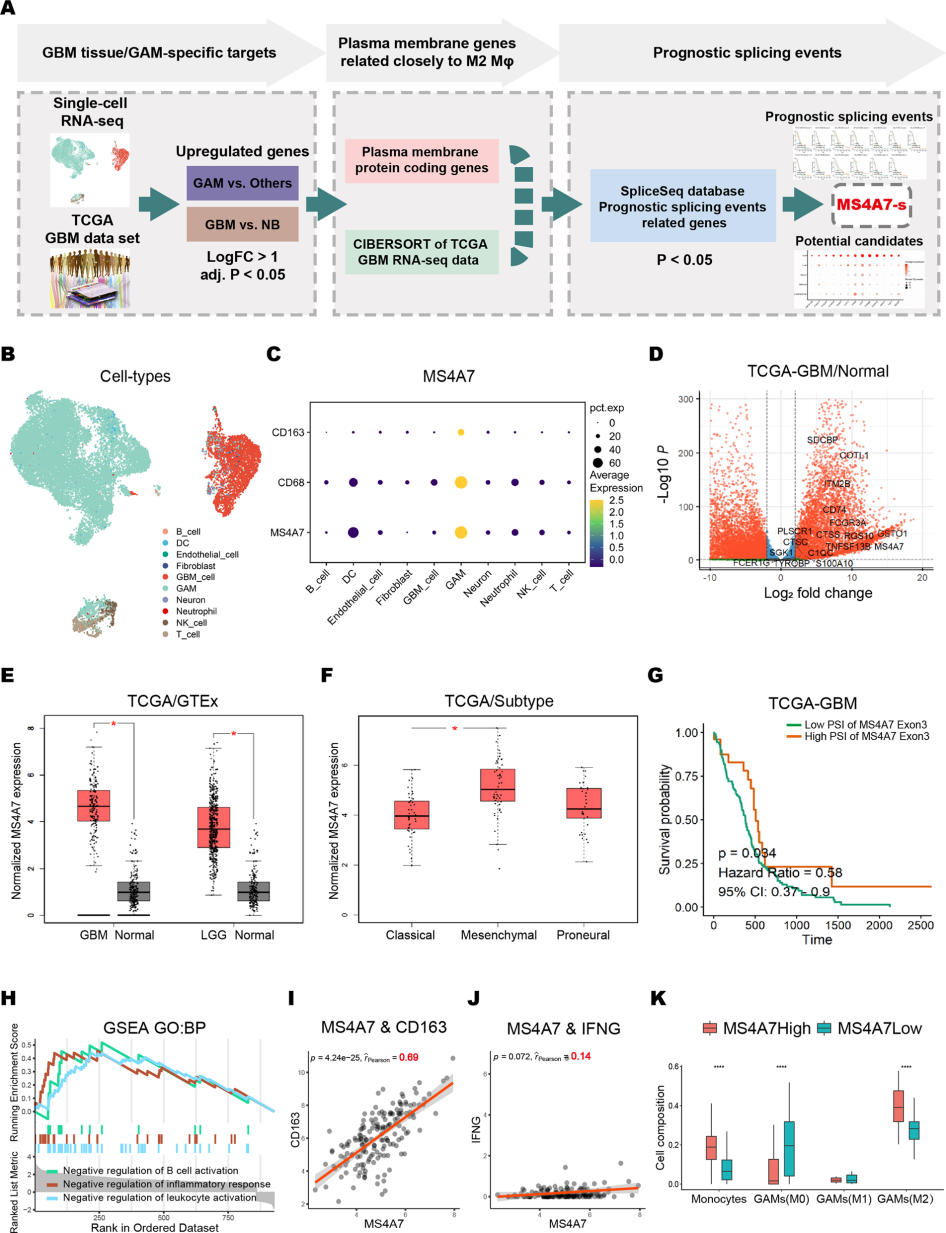
Expression of MS4A7 in GAMs of GBM tissue and its potential functions. **A** Schematic illustration of comprehensive strategies for identifying the target membrane protein. **B** UMAP plot shows different clusters of cells from scRNA-seq data. **C** Dot-plot of MS4A7, CD68 and CD163 expression levels among different types of cells. **D** Volcano plot of targeted membrane proteins upregulated in GBM tissues against normal tissues of TCGA GBM samples (adjusted *P*-value < 0.05 and log_2_fold change ≥ 1). **E** Box plot shows the mRNA expression level of MS4A7 between TCGA glioma samples (GBM and LGG) and GTEx normal brain tissues. **F** Box plot shows the mRNA expression level of MS4A7 among classical, mesenchymal and proneural subtypes of GBM. **G** Kaplan–Meier survival curves for GBM patients stratified by the PSI of MS4A7 exon 3 based on the clinical information from TCGA. **H** Enrichment plots of the signaling pathways up-regulated in the MS4A7-high expression phenotype from GSEA based on the TCGA-GBM dataset. **I**, **J** The correlation of MS4A7 with CD163 and IFNG mRNA expression was analyzed with Pearson’s test. Data were obtained from the TCGA databases. **K** Box plot shows the results of immunocellular component analysis based on TCGA GBM data using the CIBERSORT algorithm. Statistical significances were analyzed by Wilcoxon test (**E**, **K**) and one-way ANOVA (**F**). *P < 0.05; ***P < 0.001

To search for the functional protein targets that may be involved in GBM malignancy, 10× Genomic scRNA-seq were performed on clinical GBM specimens and paired adjacent normal brain tissues from four patients. Cells in the tissues were clustered and classified into different types according to the manufacturer's protocol of the R package Seurat and SingleR (Fig. 1A, B) [28, 29].

The Seurat “FindMarkers” function was used to identify the signature genes of every cell type. To search for specific molecules that facilitate targeted therapies, cell type-specific genes from scRNA-seq data, GBM-enriched genes analyzed from TCGA bulk RNA-seq data, prognostic splicing events identified from SpliceSeq data, and selection of genes encoding membrane proteins, led us to a final list of 13 genes that specifically express in GAMs. (Fig. 1A; Additional file 1: Fig. S1A, B). As shown in Additional file 1: Fig. S1C, except for MS4A7 and RGS10, the percent spliced in index (PSI) of prognostic splicing events for these genes was too close to 1 (ES) or 0 (AP/AT), reflecting the low frequency of their occurrence. Since the RGS10 protein is located mainly on the inner side of the plasma membrane and does not have an extracellular segment that is essential for targeted therapy, the short isoform MS4A7-s, generated by the prognostic splicing event, ES of MS4A7 exon 3, was finally selected, which may play a critical role in GBM TME.

As shown in Fig. 1C and Additional file 1: S1D, E, compared to adjacent normal brain tissue, MS4A7 was typically enriched in GAMs within the tumor mass, with close relation to macrophage/microglia marker CD68 and M2 marker CD163. As shown in Fig. 1D, differential expression analysis and a volcano plot showed that MS4A7 was remarkably upregulated in GBM as compared to normal brain samples from TCGA database.

Combining TCGA, GEO (GSE147352) and GTEx data, we found that expression of MS4A7 was higher in glioma samples than that in normal regions and positively correlated with glioma grading (Fig. 1E; Additional file 1: Fig. S2A). In addition, a pan-cancer analysis manifested that MS4A7 mRNA levels were widely elevated across numerous cancers, including esophageal carcinoma (ESCA), kidney chromophobe (KICH), acute myeloid leukemia (LAML), kidney renal clear cell carcinoma (KIRC), pancreatic adenocarcinoma (PAAD) and skin cutaneous melanoma (SKCM) (Additional file 1: Fig. S2B). Furthermore, among the three subtypes of GBM, the mesenchymal subtype had the highest level of MS4A7 expression (Fig. 1F). As shown in Fig. 1G, MS4A7 exon 3 skipping indicated a significantly poor prognosis for GBM patients. These data suggest that MS4A7-s may play a crucial role in determining the malignant phenotype of GBM tumors.

To explore the possible functions of MS4A7 in GBM, based on TCGA database, the 168 GBM samples were sorted by MS4A7 expression level from highest to lowest and the high or low MS4A7 expression was defined as a value in the top quartile or bottom quartile of the set, respectively. By performing gene set enrichment analysis (GSEA) between these two groups, we found out that MS4A7 expression is significantly negatively related to inflammatory response, leukocyte activation and B cell activation, which suggest its anti-inflammatory functions in GAMs (Fig. 1H). The normalized enrichment score for inflammatory response, leukocyte activation, and B cell activation were 2.06, 2.36 and 1.86. The running enrichment score for them were 0.46, 0.44 and 0.52. The *P*-values for them were 0.0018, 2.91E−05 and 0.0059. As M2 macrophages have an anti-inflammatory effect, Pearson correlation coefficient *R* between MS4A7 expression and the expression of M1/M2 macrophage markers, CD163, IL10, MSR1 (M2) and IFNG (M1) were analyzed. Intriguingly, either within GBM or glioma, MS4A7 has a strong positive correlation with CD163 (Fig. 1I, *R* = 0.69, *P* < 0.05; Additional file 1: Fig. S2E, *R* = 0.75, *P* < 0.05), IL-10 (Additional file 1: Fig. S2F, *R* = 0.79, *P* < 0.05; Additional file 1: Fig. S2G, *R* = 0.78, *P* < 0.05) and MSR1 (Additional file 1: Fig. S2H, *R* = 0.86, *P* < 0.05, Additional file 1: Fig. S2I, *R* = 0.83, *P* < 0.05), and an extremely weak correlation with IFNG (Fig. 1J, *R* = 0.14, *P* > 0.05, Additional file 1: Fig. S2J, *R* = 0.068, *P* > 0.05). Moreover, an immunocellular component analysis based on TCGA GBM data using the CIBERSORT algorithm were performed between the two groups with high and low MS4A7 expression, demonstrated a significant increase of M2 GAMs in high MS4A7 expression group. In addition, as shown in Additional file 1: Fig. S3A, pan-cancer analysis based on TCGA RNA-seq datasets showed that among ESCA, KICH, LAML, KIRC, PAAD and SKCM, only in ESCA high mRNA levels of MS4A7 was a predictor of poor outcome (*a* < 0.05). As for the correlation coefficient with M1/M2 markers, except for KICH, MS4A7 expression had a significant correlation with CD163 (Additional file 1: Fig. S3B). On the other hand, in KICH and LAML, MS4A7 expression did not have a significant correlation with IFNG (Additional file 1: Fig. S3C). These results indicate that the role of MS4A7 in a variety of tumor microenvironments and the impact of its expression on patient prognosis are almost consistent with the results of GBM tumors, and MS4A7 is an important factor associated with the suppression of macrophage inflammatory responses.

### GBM tissues predominantly express MS4A7-s isoform, a predictor of outcome in GBM patients

**Fig. 2:**
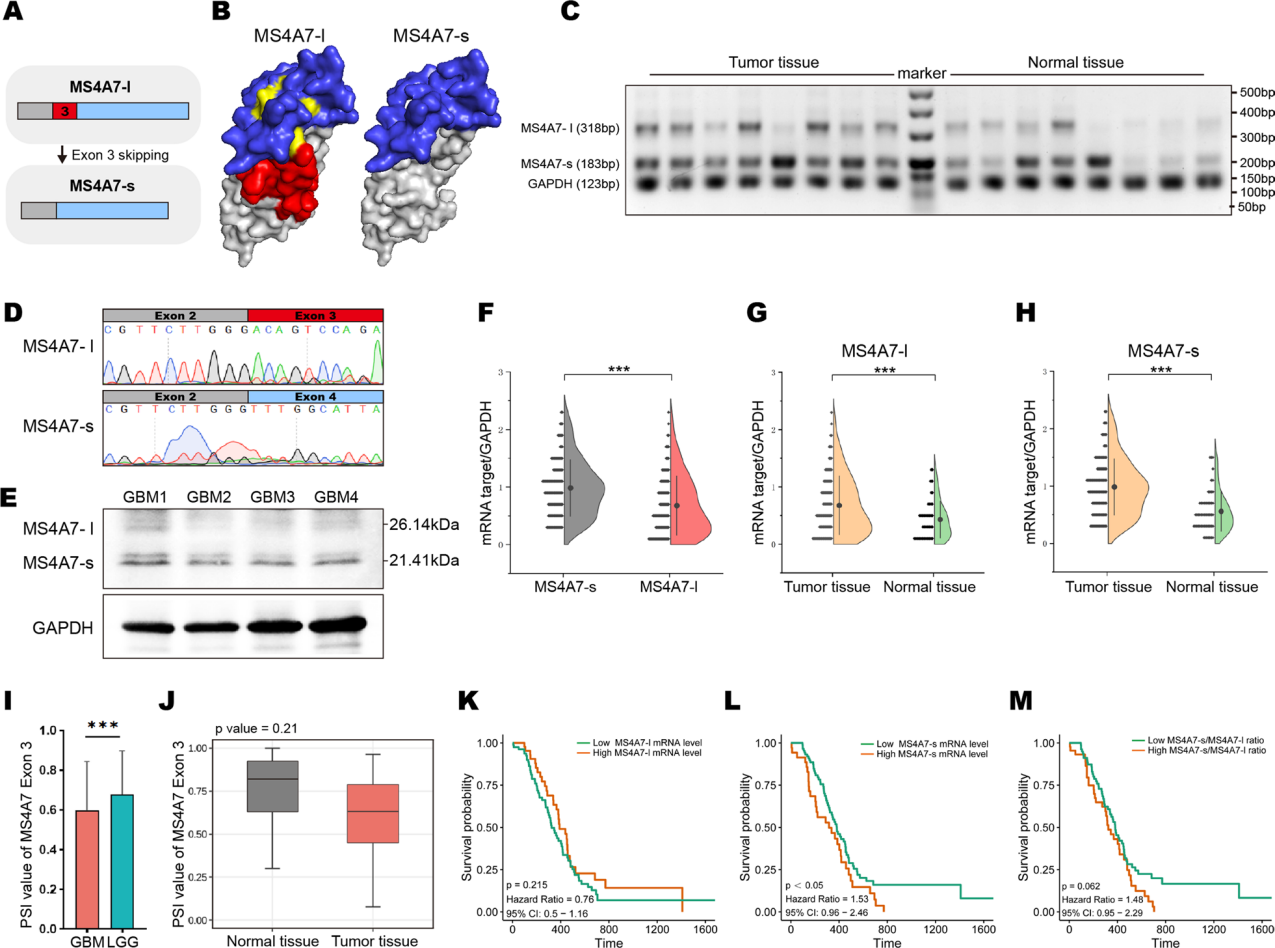
Expression of MS4A7 isoforms in GBM tissues and its clinical relevance. **A** A representation of the MS4A7-l and MS4A7-s isoforms. The alternative exon 3 of MS4A7 is in red. **B** The three-dimensional structure of MS4A7-l and MS4A7-s protein isoforms predicted by Phyre2 and plotted with PyMOL Molecular Graphics System (version 2.0 Schrodinger, LCC). **C** RT-PCR analysis of the MS4A7-l and MS4A7-s isoform mRNA levels in GBM tissues and adjacent normal tissues. **D** A representation of Sanger sequencing verifies the presence of MS4A7-l and MS4A7-s mRNA isoforms. **E** Western blotting analysis of MS4A7-l (26.14 kDa) and MS4A7-s (21.41 kDa) protein isoforms in GBM tissues. **F** Violin plot shows the relative mRNA levels of MS4A7-l and MS4A7-s isoform in GBM tissues. **G**, **H** Violin plot shows the mRNA levels MS4A7-l and MS4A7-s isoform in GBM tissues (*n* = 123) and adjacent normal brain tissues (*n* = 54). **I** Quantitative histograms shows the PSI of MS4A7 exon 3 of GBM and LGG based on the DoChaP database. **J** Box plot shows the PSI of MS4A7 exon 3 of GBM tumor tissues and the normal brain tissues based on the DoChaP database. **K**–**M** Kaplan–Meier survival curves for our GBM cohort stratified by the expression levels of MS4A7-l, MS4A7-s and MS4A7-s/MS4A7-l ratio based on the RT-PCR analysis. Statistical significances were analyzed by Student’s t test (**F**–**J**). ***P < 0.001

With or without exon 3 skipping alternative splicing, the MS4A7 gene is capable of encoding two types of transcripts (transcript 1 and 3 with exon 3 in; transcript 2 and 4 with exon 3 out), subsequently translated into so-called protein isoforms MS4A7-l and MS4A7-s, respectively (Fig. 2A). The three-dimensional structures of MS4A7-l and MS4A7-s protein isoforms were predicted by Phyre2 and plotted with PyMOL Molecular Graphics System (version 2.0 Schrodinger, LCC), based on the aa sequence of the exon-coding protein regions. As shown by three-dimensional schematic in Fig. 2B, aa50-94 (in red) coded by exon 3 of MS4A7 gene, is predicted to contain the first extracellular loop (in yellow) from N terminal of MS4A7 full-length protein (MS4A7-l), but is absent in isoform MS4A7-s [30]. Extrapolating from the three-dimensional structure, the lack of the first extracellular loop may lead to the generation of new molecule binding sites on the second extracellular loop (in blue), which suggests that MS4A7-s may play a distinct role in signal transduction relative to the full-length MS4A7-l protein. As MS4A7 isoforms’ functions and potential clinical significance have not been elucidated in any biological system, we next analyzed the presence and prognostic significance of MS4A7-s and MS4A7-l mRNA expression in 123 GBM tissues and 54 adjacent normal tissues. To simultaneously identify these two kinds of MS4A7 isoforms in tissues, RT-PCR using the special primers flanking exon 3 of MS4A7 gene was applied. Notably, RT-PCR followed by agarose gel electrophoresis yielded a 318-bp amplification product and a 183-bp one, respectively (Fig. 2C; Additional file 1: Fig. S4A), which were confirmed by Sanger sequencing (Fig. 2D). As shown in Fig. 2E, western blotting of GBM tissue total proteins identified two MS4A7 protein isoforms weighted 26.14 kDa and 21.41 kDa, respectively. To determine which isoform is the predominant form of MS4A7 transcripts, we assessed the expression of MS4A7-s and MS4A7-l mRNA. MS4A7-s levels were approximately 1.4-fold more than MS4A7-l in GBM tissues (Fig. 2C, F).

Moreover, both MS4A7-s and MS4A7-l were significantly elevated in GBM samples as compared with those in the normal brain tissues (Fig. 2G, H). We also analyzed whether the PSI of MS4A7 exon 3 differed between GBM, low-grade glioma (LGG), and normal brain using the Domain Change Presenter (DoChaP, https://dochap.bgu.ac.il/). Interestingly, LGG has an elevated PSI of exon 3 compared with GBM (Fig. 2I). Although not significantly, the PSI of MS4A7 exon 3 was slightly higher in normal brain tissue than that of GBM (Fig. 2J). These results suggested that exon 3 skipping in MS4A7 may occur more frequently in the GBM compared to the LGG. As shown in Additional file 1: Fig. S4B, the ratio of MS4A7-s’ expression levels to the sum of these two isoforms’ expression levels was not significantly different between tumor tissue and adjacent normal brain tissue. As exon 3 skipping of MS4A7 indicated a poor prognosis of GBM patients, these suggest that MS4A7 isoforms’ expression levels have potential clinical significance. Tracing back to the 123 patients’ clinical information, Kaplan–Meier analysis estimates revealed that MS4A7-l mRNA expression was not significantly associated with patients’ overall survival (OS) (Fig. 2K; *P* = 0.215); high MS4A7-s mRNA expression was a strong indicator of shortened OS (Fig. 2L; *P* = 0.0499); MS4A7-s/MS4A7-l ratio is inversely correlated with patient OS (Fig. 2M; *P* = 0.062), indicating that active alternative splicing product MS4A7-s is oncogenic in GBM.

### GAM MS4A7-s is important for the malignant phenotype of GBM cells in vitro

**Fig. 3:**
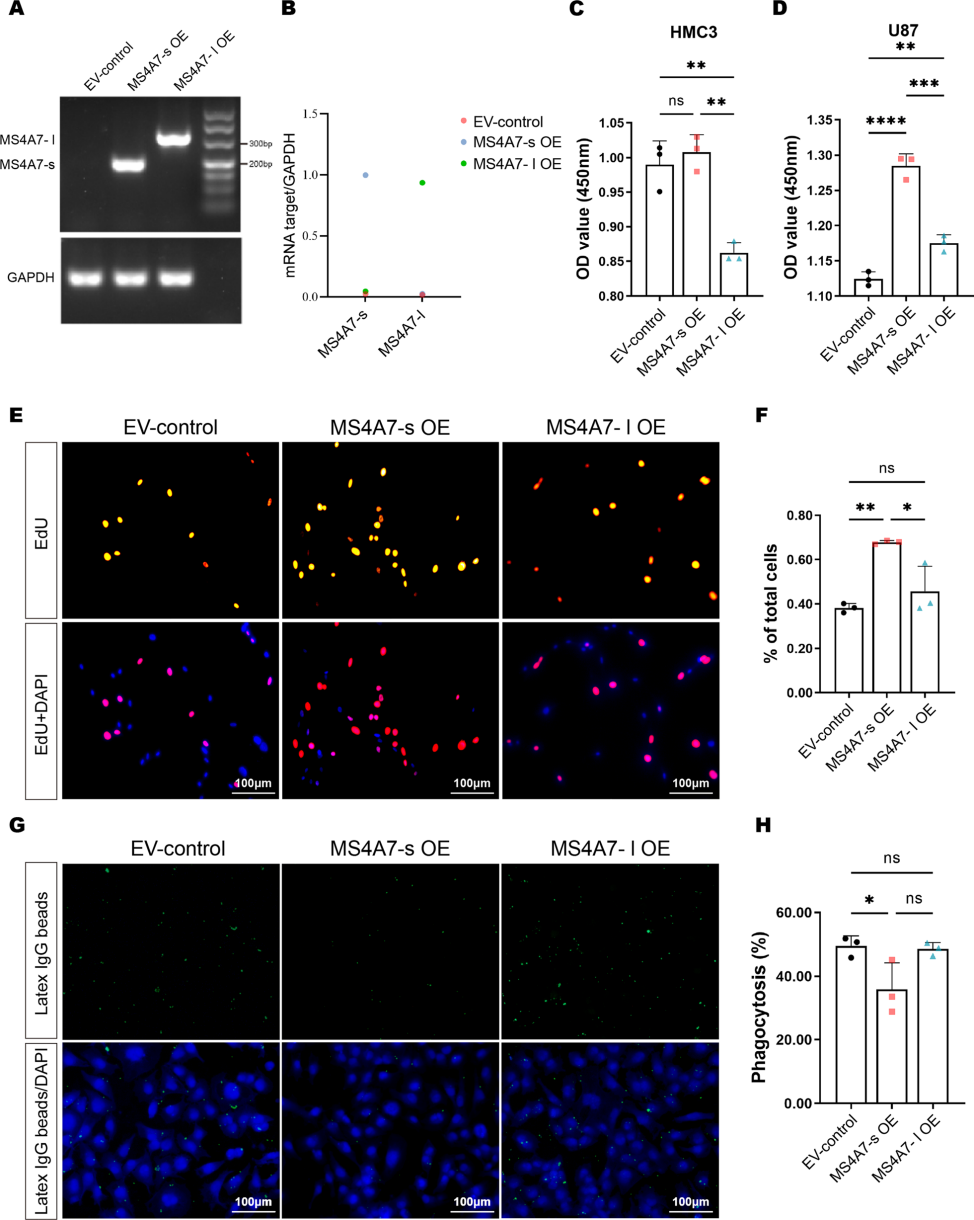
The biological functions of MS4A7-s on GAMs and co-cultured GBM cells proved by in vitro experiments. **A**, **B** RT-PCR with dot-plot statistical chart verifies MS4A7 isoforms expression in the empty vehicle-control (EV-control), MS4A7-s overexpression (MS4A7-s OE) and MS4A7-l overexpression (MS4A7-l OE) HMC3 cells. **C** HMC3 cell viability was measured by a CCK8 assay after MS4A7-s OE and MS4A7-l OE compared with the EV-control group on day 5. **D** U87MG cell viability was measured by a CCK8 assay after co-cultured with EV-control, MS4A7-s OE and MS4A7-l OE HMC3 cells on day 3. **E** EdU incorporation assay was used to detect the proliferation ability of co-cultured U87MG cells. **F** Quantitative histograms of EdU incorporation assay. **G** Phagocytosis assay by co-culturing the EV-control, MS4A7-s OE and MS4A7-l OE HMC3 cells with latex IgG beads. **H** Quantitative histograms of phagocytosis assay. Data are presented as mean ± SD (*n* = 3/group). Statistical significances were analyzed by one-way ANOVA (**C**, **D**, **F**, **H**). *P < 0.05; **P < 0.01; ***P < 0.001; ****P < 0.0001

Prompted by these results, we further investigated whether GAMs-derived MS4A7 isoforms MS4A7-s and MS4A7-l were essential for the malignant phenotype of GBM cells in vitro. We first utilized lentivirus to construct MS4A7-s and MS4A7-l overexpressed (MS4A7-s OE and MS4A7-l OE) HMC3 cells, which were validated by RT-PCR (Fig. 3A, B). We next examined the effect of overexpressed MS4A7-s and MS4A7-l on the HMC3 cell itself. As shown in Fig. 3C, by performing a Cell Counting Kit-8 (CCK8) assay, we found that MS4A7-l OE substantially decreased the number of HMC3 cells as compared to the control or the MS4A7-s OE group, indicating that MS4A7-l hinders GAM proliferation, while MS4A7-s has no apparent effect on this.

We next explored the biological function of lentivirus-infected HMC3 cells on co-cultured GBM cell line U87MG. To this end, U87MG cells were incubated with conditioned medium (CM) from different groups of HMC3 cells for 3 days, and cell proliferation was assessed by a CCK8 assay. As shown in Fig. 3D, the CM for MS4A7-s OE HMC3 cells possesses a stronger pro-U87 cell proliferation effect relative to that of control cells and MS4A7-l OE cells. To further test this finding, a Boyden chamber-based co-culture system were deployed, followed by a 5-ethynyl-2′-deoxyuridine (EdU) assay to further characterize the MS4A7-s OE HMC3 cells’ pro-proliferative effects on U87MG. As shown in Fig. 3E and F, consistent with the CCK8 assay, MS4A7-s OE HMC3 cells were proliferation-inducing to co-cultured U87MG cells compared with the control group and the MS4A7-l OE group.

We also performed an in vitro bead-based phagocytosis assay to examine the effect of GAMs’ MS4A7 isoforms on GBM cells. As shown in Fig. 3G, MS4A7-s OE significantly diminished the phagocytotic activity with a decreased percentage of identification of HMC3 cells with intracellular latex beads compared to the control group, whereas MS4A7-l OE did not (Fig. 3H). Together, these results indicate that the MS4A7 gene plays a pro-oncogenic role in the GAM and even in the GBM TME, mainly in the form of MS4A7-s.

### Overexpression of MS4A7-s in GAMs enhances the growth of GBM xenografts

**Fig. 4:**
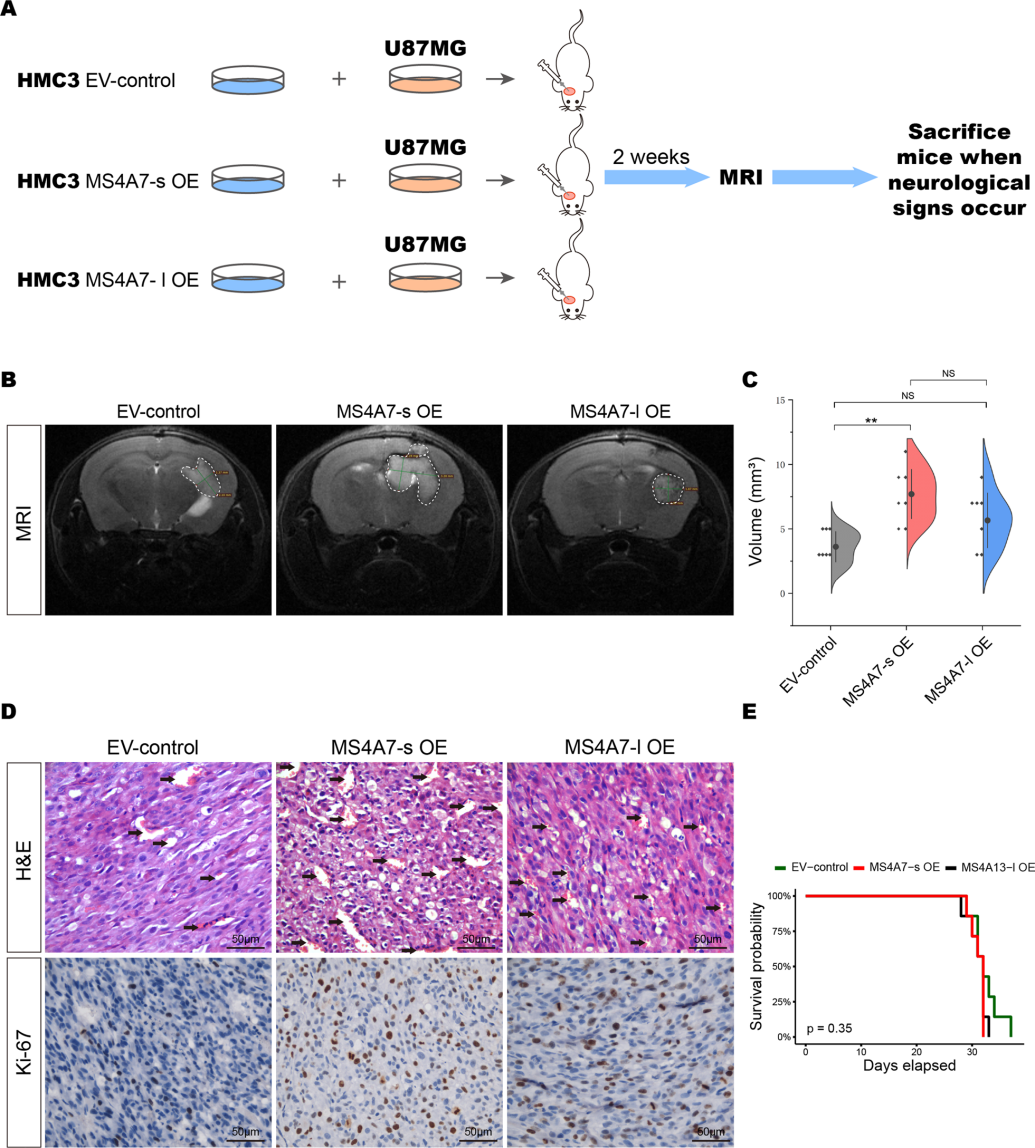
Intracranial xenograft formation assay to clarify the tumor-promoting function of MS4A7-s in vivo. **A** The flow diagram of intracranial tumorigenesis experiment. HMC3 cells of EV-control, MS4A7-s OE and MS4A7-l OE group were co-injected with U87MG cells with a ratio of 3:4 into the brains of BALB/c immunodeficient (SCID) mice (*n* = 7 in each group). MRI was applied to assess the volume of tumors 2 weeks after injection and euthanasia was deployed when neurological signs occurred. **B** Representative MRI images show the tumors of EV-control, MS4A7-s OE and MS4A7-l OE group, respectively. **C** Quantitative violin plot of xenograft formation assay (*n* = 7 in each group). **D** H&E and Ki-67 IHC staining of the slides of tumor-bearing mice in EV-control, MS4A7-s OE and MS4A7-l OE group. **E** Survival curves of intracranial tumor-bearing mice co-injected with U87MG and EV-control, MS4A7-s OE and MS4A7-l OE HMC3 cells. Technical replicates were performed for each group as indicated. Statistical significances were analyzed by one-way ANOVA (**C**). *ns* not significant; **P < 0.01

To determine the roles of GAMs-derived MS4A7-s in GBM tumorigenesis in mice bearing intracranial xenografts model, U87MG cells were co-injected with control, MS4A7-l OE or MS4A7-s OE HMC3 cells into the brains of BALB/c immunodeficient (SCID) mice (n = 7 in each group) (Fig. 4A). MRI was performed 14 days after co-injection. As shown in Fig. 4B and Additional file 1: Fig. S5, tumor volumes were significantly larger in the group with MS4A7-s OE HMC3 cells co-injected, compared to the control group (Fig. 4C). On the other hand, volumes of tumors with MS4A7-l OE HMC3 cells co-injected were not significantly different compared to the control group (Fig. 4C). This result, which is following the in vitro proliferation assays, demonstrates that GAM-derived MS4A7-s, rather than MS4A7-l, is crucially important for GBM tumor tumorigenesis. Furthermore, with hematoxylin–eosin (H&E) staining of xenografts, we found more blood vessels in the tumors of mice in the MS4A7-s OE group compared to the control and MS4A7-l OE groups (Fig. 4D, top), which may promote tumor growth. Meanwhile, the proliferation rate of tumor cells was examined by Ki-67 staining, and the MS4A7-s OE group had the highest growth rate (20.28%), compared to the MS4A7-l OE (10.65%) and the control group (8.22%, Fig. 4D, bottom). Unfortunately, as shown in Fig. 4E, there was no significant difference in survival time among the three groups of mice, probably because the re-education of GAMs by the GBM TME contributed to their pro-tumorigenic activation, resulting in a relatively rapid growth for all these tumors and making it difficult to reflect the difference in survival.

### Overexpression of MS4A7-s activates PI3K/AKT/GSK3β pathway and leads to M2 polarization of GAMs

**Fig. 5:**
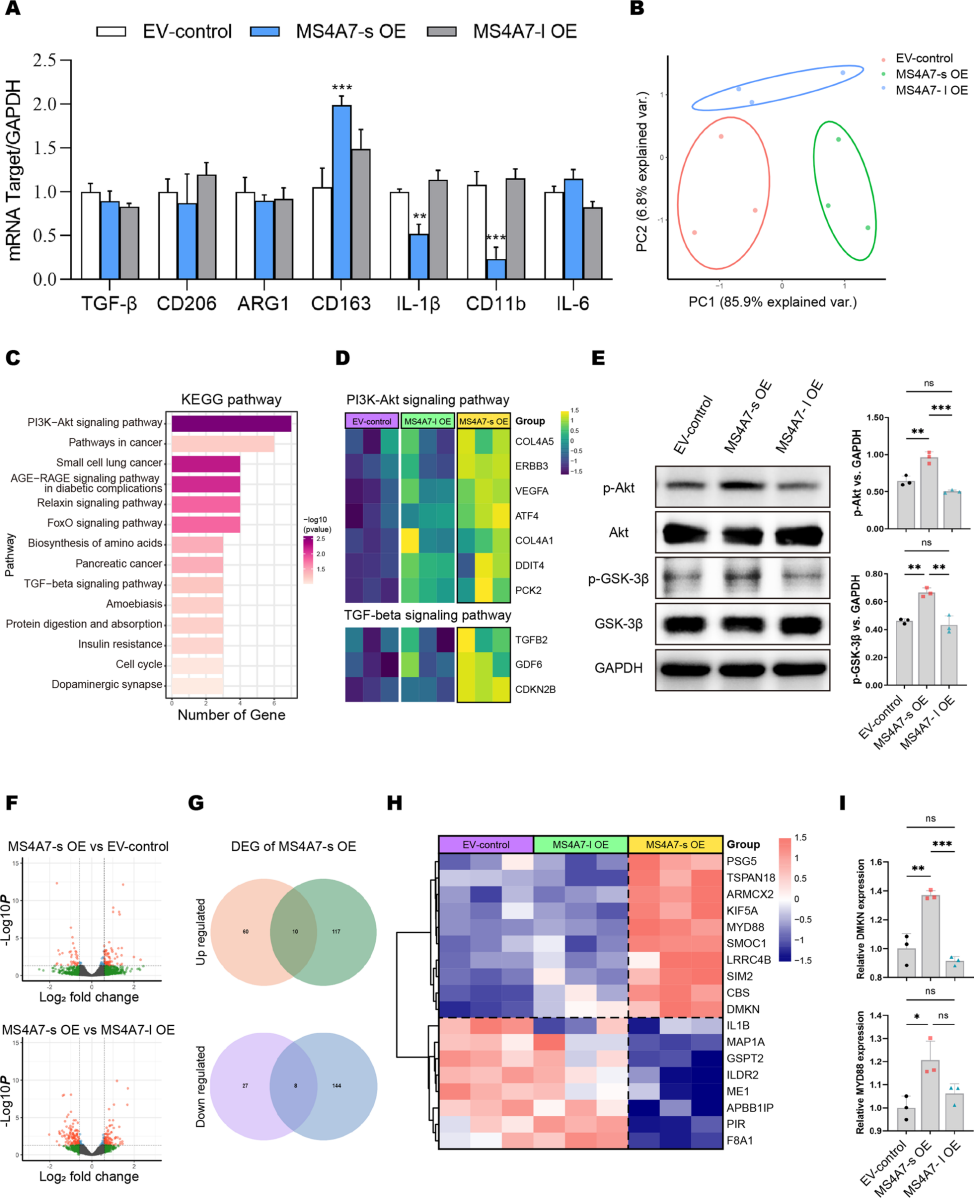
Transcriptome profile and signaling pathway activity of EV-control, MS4A7-s OE and MS4A7-l OE HMC3 cells. **A** Quantitative histograms of M1 and M2 markers detected by qRT-PCR in EV-control, MS4A7-s OE and MS4A7-l OE HMC3 cells. **B** PCA plot of RNA-seq data of EV-control, MS4A7-s OE and MS4A7-l OE HMC3 cells. **C** KEGG pathway enrichment analysis of up-regulated genes in MS4A7-s OE HMC3 cells compared to EV-control group. **D** Heatmap of MS4A7-s OE group up-regulated genes that are enriched in PI3K-Akt signaling pathway and TGF-beta signaling pathway. **E** Western blotting analysis of p-AKT, AKT, p-GSK-3β and GSK-3β expression levels in EV-control, MS4A7-s OE and MS4A7-l OE HMC3 cells. GAPDH was used for normalization. **F** Volcano plots of DEGs between MS4A7-s OE and EV-control group and DEGs between MS4A7-l OE and EV-control group. **G** Venn diagrams depict overlaps of differential expressing genes in MS4A7-s OE HMC3 cells. **H** Heatmap depicts overlaps of differential expressing genes in EV-control, MS4A7-s OE and MS4A7-l OE HMC3 cells. (I) Quantitative histograms of DMKN and MYD88 mRNA expression level with qRT-PCR. GAPDH was used for normalization. Data are presented as mean ± SD (*n* = 3/group). Statistical significances were analyzed by one-way ANOVA (**A**, **I**). ns not significant, *P < 0.05; **P < 0.01; ***P < 0.001

Because MS4A7 expression can be elevated by anti-inflammation factors and is closely associated with macrophage M2 polarization with bioinformatic analyses, we hypothesized that MS4A7 isoforms play crucial roles in the polarization phenotype of GAMs. To this end, we applied qRT-PCR to measure the expression level of IL-1β, CD11b and IL6 (M1 parameters), and TGF-β, CD206, ARG1 and CD163 (M2 parameters) among different groups of HMC3 cells. As shown in Fig. 5A, compared to the control group and the MS4A7-l OE group, CD163 was significantly up-regulated in the MS4A7-s OE group, with IL-1β and CD11b down-regulated remarkably, which suggest that MS4A7-s can promote macrophage M2 polarization and block M1 associated pro-inflammation activity. To further investigate the mechanism by which MS4A7-s affects the polarization phenotype of GAMs and exerts tumor-promoting effects, we extracted total RNA from control, MS4A7-s OE and MS4A7-l OE groups of HMC3 cells for RNA-seq. After pre-processing of RNA-seq data, we first performed principal component analysis (PCA) on those three groups of HMC3 cells. Interestingly, it was apparent from Fig. 5B that the MS4A7-s OE group was strikingly distinct from the control group in PC1 that had a large explainable variation (85.9%). Nevertheless, the MS4A7-l OE group differed from the other two groups mainly in PC2 that has a minor explainable variation (6.8%). This result together with the above-mentioned data further indicates that the isoform MS4A7-s plays a major role among the expression products of the MS4A7 gene. To explore the potential signaling pathways that are active in the MS4A7-s OE GAMs, we used R package DESeq2 to get the up-regulated differential expressing genes (DEGs) in the MS4A7-s OE group compared to the control group followed by the KEGG pathway enrichment analysis [31, 32]. As shown in Fig. 5C, D and Additional file 1: Fig. S6A, the PI3K–Akt signaling pathway, angiogenesis pathway, and TGF-beta signaling pathway were up-regulated in the MS4A7-s OE group, compared to the control group. Previous studies have shown that macrophage MSR1 promoted M2-like polarization by activating PI3K/AKT/GSK3β pathway [33]. Moreover, M2 macrophages induce the malignant phenotype of cancer cells through TGF-beta signaling [34, 35]. To confirm these findings, we verified the activation of the PI3K/AKT/GSK3β pathway in MS4A7-s OE cells using western blotting of Akt (phosphorylated at position Ser 473) and GSK-3β (phosphorylated at position Ser 9) phosphorylation levels in three groups of HMC3 cells (Fig. 5E). Additionally, MAPK signaling pathway and cell migration were upregulated in the MS4A7-l OE group compared to the control (Additional file 1: Fig. S6B, C).

To more clearly elucidate the unique and important role of MS4A7-s isoform, we obtained the DEGs between the MS4A7-s OE group and the MS4A7-l OE group (Fig. 5F), and took the intersection of them with the DEGs between the MS4A7-s OE group and the control group as up-regulated or down-regulated, respectively (Fig. 5G). As shown in Fig. 5H, there were 10 up-regulated genes and 8 down-regulated genes in MS4A7-s OE HMC3 cells, and the up-regulated genes are listed in Table 1. Verified by qRT-PCR, DMKN and MYD88 were significantly elevated in the MS4A7-s OE group, compared to the control group (Fig. 5I). Previous studies showed that DMKN contributed to epithelial–mesenchymal transition in pancreatic cancer. Moreover, MyD88 in myofibroblasts can promote macrophage M2 polarization. All these results are consistent with the tumor-promoting and effects on the polarization of MS4A7-s, further supporting our observations.

**Table 1:** Gene profiling of MS4A7-s OE HMC3 cells

Symbol	Gene name	Ensembl Gene ID	Fold change (vs. EV-control)	MF/BP/CC (Gene ontology)
CBS	Cystathionine beta-synthase	ENSG00000160200	2.80	Cystathionine beta-synthase activity
LRRC4B	Leucine-rich repeat containing 4B	ENSG00000131409	2.68	Signaling receptor binding
DMKN	Dermokine	ENSG00000161249	2.45	Cornified envelope assembly
SMOC1	SPARC related modular calcium binding 1	ENSG00000198732	2.39	Extracellular matrix binding, calcium ion binding
KIF5A	Kinesin family member 5A	ENSG00000155980	2.32	Cytoskeletal motor activity
SIM2	SIM bHLH transcription factor 2	ENSG00000159263	2.03	DNA-binding transcription factor activity, RNA polymerase II-specific
MYD88	MYD88 innate immune signal transduction adaptor	ENSG00000172936	1.92	Interleukin-1 receptor binding
PSG5	Pregnancy specific beta-1-glycoprotein 5	ENSG00000204941	1.80	Heterophilic cell–cell adhesion via plasma membrane cell adhesion molecules
ARMCX2	Armadillo repeat containing X-linked 2	ENSG00000184867	1.77	Integral component of membrane
TSPAN18	Tetraspanin 18	ENSG00000157570	1.55	Ion channel activity

Fold induction of genes up-regulated in MS4A7-s OE HMC3 cells versus EV-control cells and MS4A7-l OE cells under normal growth conditions. Only genes with a fold change of 1.5 are presented

## Discussion

The TME of GBM contains many different non-cancerous types of cells, including immune cells, endothelial cells, pericytes, and fibroblasts [9, 36]. Our previous studies suggested that different AS products may have distinct functions in GBM progression and thus contribute to intratumoral heterogeneity [23, 24]. AS products are present not only in tumor cells, but also in other cells of TME. After screening for membrane proteins that were significantly elevated in the cell surface of TME, we performed survival analysis of splicing events based on the SpliceSeq database, and finally identified the prognostic splicing event, ES of MS4A7 exon 3 and its expression product isoform MS4A7-s, potentially highly enriched in TME and is closely associated with a poor prognosis of patients [37].

MS4A7 belongs to the MS4A family, which includes 18 proteins that have a similar four-transmembrane structure, with N and C terminals protruding into the cytoplasm, and two extracellular loop structures between the transmembrane regions [38, 39]. The extracellular loop structures of this family of proteins tend to have low sequence identity and can regulate cell signaling as components of receptor complexes [40]. It has been predicted that MS4A7 could encode two different protein isoforms with lengths of 240aa (MS4A7-1, full length) and 195aa (MS4A7-s), which was confirmed in our study [38]. Although MS4A7 gene can be transcribed into four transcripts, they produce only 2 protein isoforms. Transcript 1 and transcript 3 have the same coding sequence (CDS) and encode the same protein isoform, MS4A7-1. Likewise, both transcript 2 and transcript 4 encode the same protein isoform, MS4A7-s. The MS4A7-s transcript is generated by alternative splicing of exon 3 skipping, compared to full-length MS4A7-l. It has been reported that 18 protein members of the human MS4A family are differentially expressed in different leukocyte subpopulations, whereas the functions of MS4A7 have never been investigated [39, 40]. MS4A7 may be involved in mature cell functions in the monocyte lineage, where it may be a component of a receptor complex involved in signal transduction [38]. To demonstrate the existence of the two splicing isoforms of MS4A7, we examined them in a large number of clinical GBM specimens at both the RNA and protein levels. These data fully identified MS4A7-s as the major expression product of the MS4A7 gene. Through an exhaustive analysis of clinical data using patient-corresponding specimens, we demonstrated that MS4A7-s, but not MS4A7-l, is a robust predictor of survival in GBM patients.

Our data show MS4A7-s is mainly expressed in GAMs. Ms4a8a (the murine homologue of human MS4A8B) expressed on macrophages has been shown to play an important role in promoting the growth of subcutaneously transplanted mammary tumors [41]. Ms4a6d, the mouse homologue of human MS4A6A, suppresses the inflammatory signature of macrophages [42]. These findings promoted us to investigate the contribution of MS4A7-s in GAMs to maintain GBM tumor growth by in vitro and in vivo experiments.

GAMs account for 30–50% of the cellular content of GBM tumor masses [36]. Once present in the TME, microglia and macrophages, which should have anti-tumor cytotoxic and phagocytic functions, will transform into GAMs, acquiring the ability to maintain tumor growth and even leading to treatment resistance [43–45]. Several studies have identified proteins such as CD47 that can cause a severe reduction in phagocytosis of GBM cells by macrophages/microglia and have led to the development of methods to block its action for treating GBM [46–48]. GAMs are usually polarized into M2 and M1 types [49, 50]. Microglia and macrophages are remarkable plastic cells that can switch from one phenotype to another [51]. With the deepening of research, it has been demonstrated that activation status rather than GAM abundance has important prognostic value [51].

For further verification, we investigated the transcriptomic changes in macrophages affected by MS4A7-s expression status. According to RNA-seq data, the KEGG signaling pathway enrichment analysis suggests that MS4A7-s is essential for M2-type polarization of macrophages due to the activation of PI3K/Akt, which was consistent with the results of past published literature [33]. Then, the phosphorylation levels of AKT and downstream GSK3β in lentivirus-infected HMC3 cells were verified using western blotting. Moreover, our RNA-seq analysis and qRT-PCR results indicate that DMKN and MYD88 were significantly upregulated when MS4A7-s was overexpressed. These are consistent with several studies which have shown that overexpression of DMKN enhances the proliferation and epithelial–mesenchymal transition of pancreatic tumor cells; MyD88 in myofibroblasts can promote macrophage M2 polarization [52, 53]. The above results are in line with the role of MS4A7-s in the M2 polarization of GAMs and GBM malignant phenotype, further supporting our observation [54].

From the perspective of protein folding or spatial structure, MS4A7-s, with an exon missing compared with MS4A7-l, achieves the polarization function of GAMs from M1 to M2. Further studies are needed to reveal the specific molecular interactions and mechanisms.

Taken together, our study used a combination of scRNA-seq and bulk RNA-seq to profile the membrane proteins of GBM TME, and combined with alternative splicing data to analyze prognostic splicing events. This study demonstrates for the first time that the expression of MS4A7 and its main expression product MS4A7-s in GAMs, as it can promote the polarization of GAMs to M2-type, is a robust predictor of poor prognosis in GBM patients with the ability to maintain the growth of GBM cells, suggesting that targeting MS4A7-s function might be a promising treatment for GBM. In this study, when screening specific molecules, the targets were restricted to the proteins localized in the cell plasma membrane, which also helped in designing MS4A7-s-based therapeutic strategies.

## Supplementary Information

**Additional file 1: Figure S1.** Candidate prognostic splicing events associated plasma membrane-located proteins screened by comprehensive analysis. (A) Kaplan–Meier survival curves for prognostic splicing events based on SpliceSeq database and TCGA GBM clinical data. (B) Dot-plot shows the expression of candidate genes among the different cell types within scRNA-seq data of GBM tissue. (C) Quantitative histograms of PSI value of candidate prognostic splicing events. Data are presented as mean ± SD. (D) UMAP plot shows different clusters of cells from GBM tissue and adjacent normal tissue scRNA-seq data. (E) Dot-plot of MS4A7 expression levels among different types of cells from GBM tissue and adjacent normal tissue scRNA-seq data. **Figure S2.** Expression pattern and relevance to the outcome of GBM patients of MS4A7 based on TCGA data and GEO dataset. (A) Box plot of expression levels of MS4A7 among GBM, LGG and normal tissues based on dataset GSE147352. (B) A Pan-cancer analysis: box plot of MS4A7 expression against corresponding GTEx normal tissues among ESCA, KICH, KIRC, LAML, PAAD and SKCM. (C) Kaplan–Meier survival curves stratified by the expression levels of MS4A7 based on a CGGA GBM dataset. (D) Kaplan–Meier survival curves stratified by the expression levels of MS4A7 based on TCGA glioma dataset. (E) The correlation of MS4A7 with CD163 mRNA expression was analyzed with Pearson’s test. Data were obtained from the TCGA glioma dataset. (F, G) The correlation of MS4A7 with IL10 mRNA expression was analyzed with Pearson’s test. Data were obtained from the TCGA GBM and glioma dataset. (H, I) The correlation of MS4A7 with MSR1 mRNA expression was analyzed with Pearson’s test. Data were obtained from the TCGA GBM and glioma dataset. (J) The correlation of MS4A7 with IFNG mRNA expression was analyzed with Pearson’s test. Data were obtained from the TCGA glioma dataset. Statistical significances were analyzed by one-way ANOVA (A) and Wilcoxon test (B). *P < 0.05, **P < 0.01, ***P < 0.001. **Figure S3.** Pan-cancer analysis of MS4A7 based on TCGA data. (A) Kaplan–Meier survival curves stratified by the expression levels of MS4A7 based on TCGA ESCA, KICH, KIRC, LAML, PAAD and SKCM datasets. (B) The correlation of MS4A7 with CD163 mRNA expression was analyzed with Pearson’s test. Data were obtained from the TCGA ESCA, KICH, KIRC, LAML, PAAD and SKCM datasets. (C) The correlation of MS4A7 with IFNG mRNA expression was analyzed with Pearson’s test. Data were obtained from the TCGA ESCA, KICH, KIRC, LAML, PAAD and SKCM datasets. **Figure S4.** RT-PCR of MS4A7 isoforms mRNA levels in GBM (n = 123) and adjacent normal brain tissues (n = 54). (A) RT-PCR of MS4A7 isoforms mRNA levels in GBM and adjacent normal brain tissues. (B) Violin plot of MS4A7-s/ (MS4A7-s + MS4A7-l) ratio between GBM tissues and adjacent normal brain tissues. Statistical significances were analyzed by Student’s t test. ns not significant. **Figure S5.** MRI images of tumors of EV-control group, MS4A7-s OE group, and MS4A7-l OE group. **Figure S6.** Enrichment analyses of up-regulated genes of MS4A7-s OE and MS4A7-l OE group HMC3 cells. (A) Up-regulated pathway of MS4A7-s OE group based on GO:BP dataset. (B) Up-regulated pathway of MS4A7-l OE group based on KEGG dataset. (C) Up-regulated pathway of MS4A7-l OE group based on GO:BP dataset.

**Additional file 2. **Clinical information of the GBM cohort.

**Additional file 3. **Primer pairs used in this study.

## Data Availability

The data sets used and analyzed during the current study are available from the corresponding author on reasonable request.

## Abbreviations

NB: Normal brain
ES: Exon skipping
AP: Alternate promoter
AT: Alternate terminator
KEGG: Kyoto Encyclopedia of Genes and Genomes
GO: Gene Ontology
BP: Biological process
MF: Molecular function
CC: Cell component
TCGA: The Cancer Genome Atlas
CGGA: Chinese Glioma Genome Atlas
LGG: Low-grade glioma
UMAP: Uniform Manifold Approximation and Projection
IHC: Immunohistochemistry
DEGs: Differentially expressed genes
GEO: Gene Expression Omnibus
