# Supplementary material for: The short isoform of MS4A7 is a novel player in glioblastoma microenvironment, M2 macrophage polarization, and tumor progression
Source: J Neuroinflammation. 2023 Mar 21;20:80. doi: 10.1186/s12974-023-02766-1 (PMC10031966; doi:10.1186/s12974-023-02766-1)
Supplement: Supplementary file 1 — Additional file 1: Figure S1. Candidate prognostic splicing events associated plasma membrane-located proteins screened by comprehensive analysis. (A) Kaplan–Meier survival curves for prognostic splicing events based on SpliceSeq database and TCGA GBM clinical data. (B) Dot-plot shows the expression of candidate genes among the different cell types within scRNA-seq data of GBM tissue. (C) Quantitative histograms of PSI value of candidate prognostic splicing events. Data are presented as mean ± SD. (D) UMAP plot shows different clusters of cells from GBM tissue and adjacent normal tissue scRNA-seq data. (E) Dot-plot of MS4A7 expression levels among different types of cells from GBM tissue and adjacent normal tissue scRNA-seq data. Figure S2. Expression pattern and relevance to the outcome of GBM patients of MS4A7 based on TCGA data and GEO dataset. (A) Box plot of expression levels of MS4A7 among GBM, LGG and normal tissues based on dataset GSE147352. (B) A Pan-cancer analysis: box plot of MS4A7 expression against corresponding GTEx normal tissues among ESCA, KICH, KIRC, LAML, PAAD and SKCM. (C) Kaplan–Meier survival curves stratified by the expression levels of MS4A7 based on a CGGA GBM dataset. (D) Kaplan–Meier survival curves stratified by the expression levels of MS4A7 based on TCGA glioma dataset. (E) The correlation of MS4A7 with CD163 mRNA expression was analyzed with Pearson’s test. Data were obtained from the TCGA glioma dataset. (F, G) The correlation of MS4A7 with IL10 mRNA expression was analyzed with Pearson’s test. Data were obtained from the TCGA GBM and glioma dataset. (H, I) The correlation of MS4A7 with MSR1 mRNA expression was analyzed with Pearson’s test. Data were obtained from the TCGA GBM and glioma dataset. (J) The correlation of MS4A7 with IFNG mRNA expression was analyzed with Pearson’s test. Data were obtained from the TCGA glioma dataset. Statistical significances were analyzed by one-way ANOVA (A) and Wilcoxon test (B). *P < 0.05, **P < 0 [file 12974_2023_2766_MOESM1_ESM.pdf]

Additional file 1

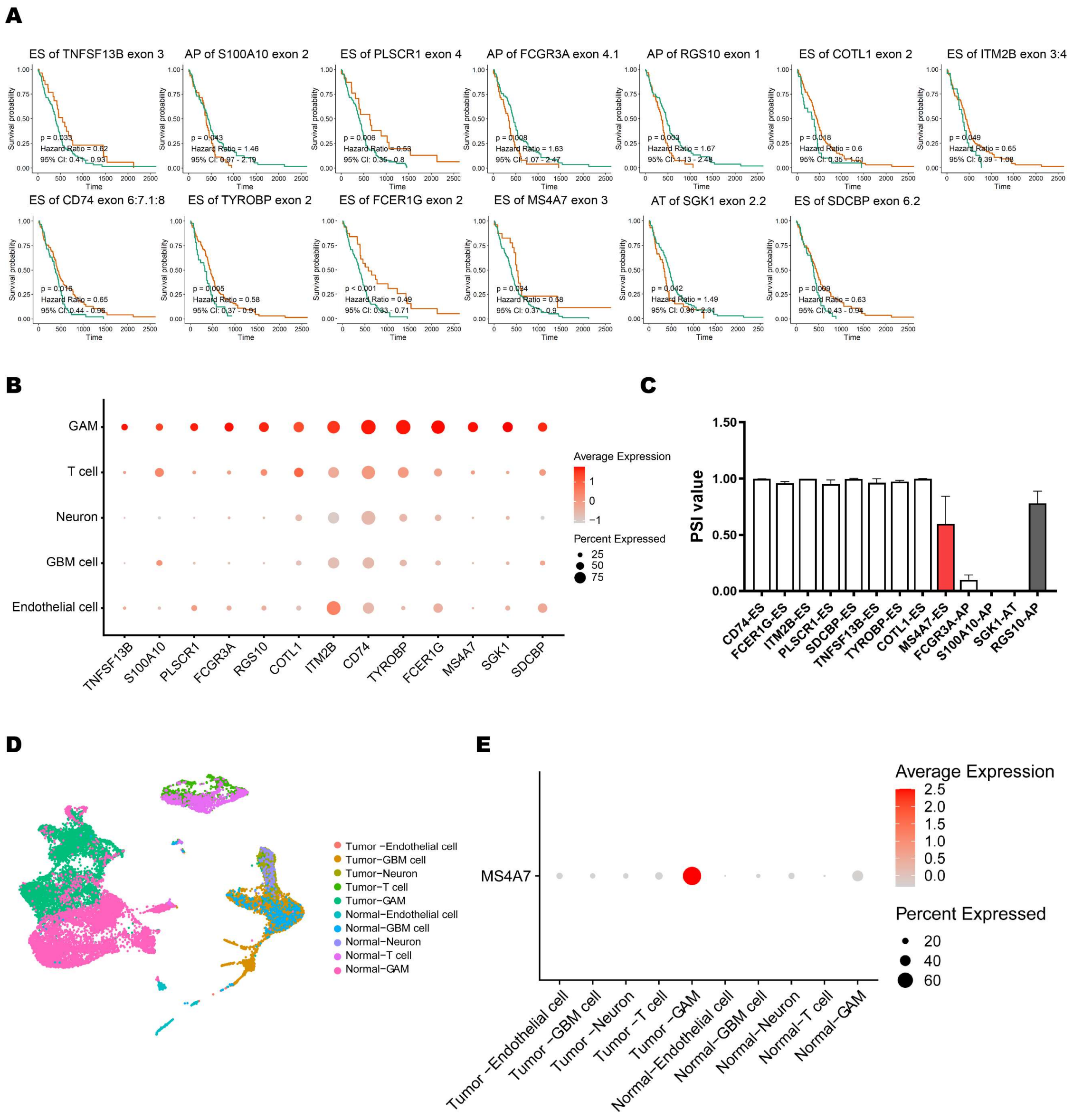

Figure S1

Candidate prognostic splicing events associated plasma membrane-located proteins screened by comprehensive analysis

(A) Kaplan–Meier survival curves for prognostic splicing events based on SpliceSeq database and TCGA GBM clinical data. (B) Dotplot shows the expression of candidate genes among the different cell types within scRNA-seq data of GBM tissue. (C) Quantitative histograms of PSI value of candidate prognostic splicing events. Data are presented as mean ± SD. (D) UMAP plot shows different clusters of cells from GBM tissue and adjacent normal tissue scRNA-seq data. (E) Dotplot of MS4A7 expression levels among different types of cells from GBM tissue and adjacent normal tissue scRNA-seq data.

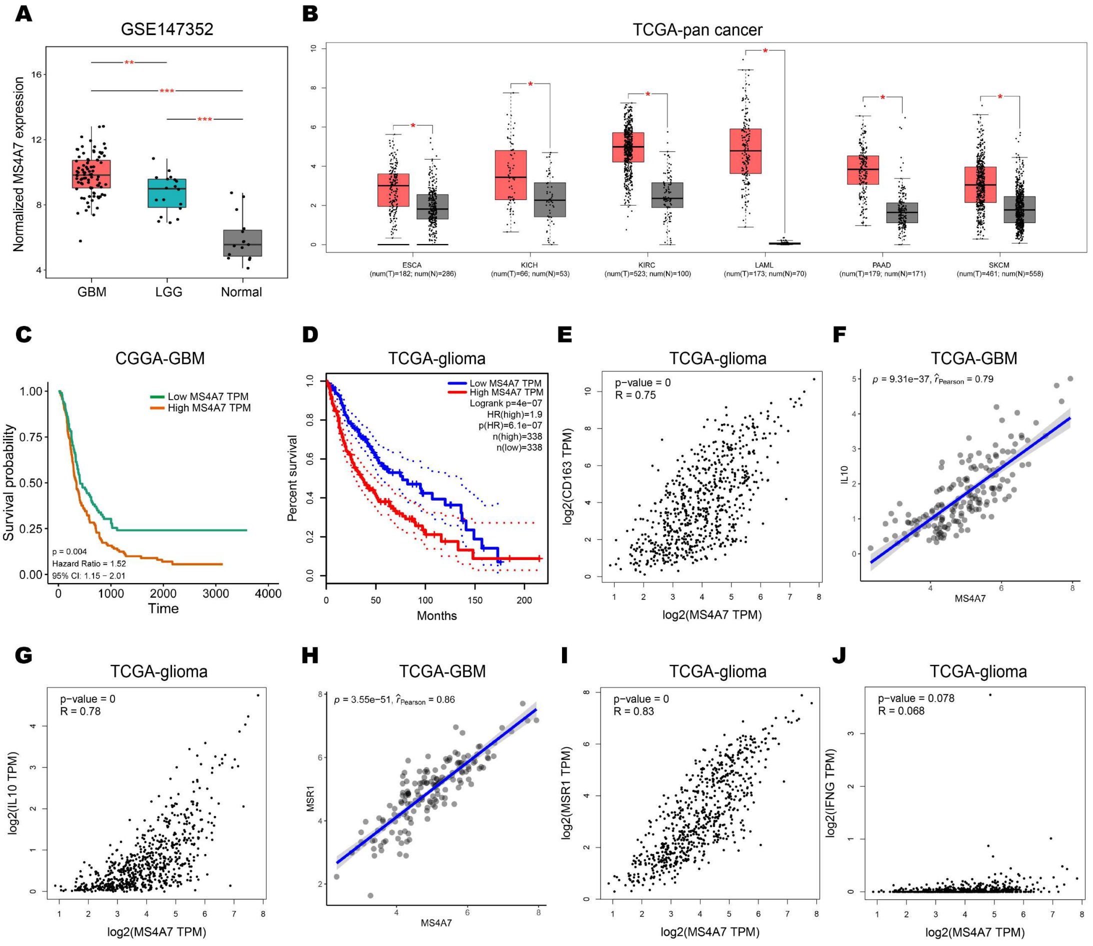

Figure S2

Expression pattern and relevance to the outcome of GBM patients of MS4A7 based on TCGA data and GEO dataset

(A) Box plot of expression levels of MS4A7 among GBM, LGG and normal tissues based on dataset GSE147352. (B) A Pan-cancer analysis: box plot of MS4A7 expression against corresponding GTEx normal tissues among ESCA, KICH, KIRC, LAML, PAAD and SKCM. (C) Kaplan–Meier survival curves stratified by the expression levels of MS4A7 based on a CGGA GBM dataset. (D) Kaplan–Meier survival curves stratified by the expression levels of MS4A7 based on TCGA glioma dataset. (E) The correlation of MS4A7 with CD163 mRNA expression was analyzed with Pearson's test. Data were obtained from the TCGA glioma dataset. (F, G) The correlation of MS4A7 with IL10 mRNA expression was analyzed with Pearson's test. Data were obtained from the TCGA GBM and glioma dataset. (H, I) The correlation of MS4A7 with MSR1 mRNA expression was analyzed with Pearson's test. Data were obtained from the TCGA GBM and glioma dataset. (J) The correlation of MS4A7 with IFNG mRNA expression was analyzed with Pearson's test. Data were obtained from the TCGA glioma dataset. Statistical significances were analyzed by one-way ANOVA (A) and Wilcoxon test (B). *P < 0.05, **P < 0.01, ***P < 0.001.

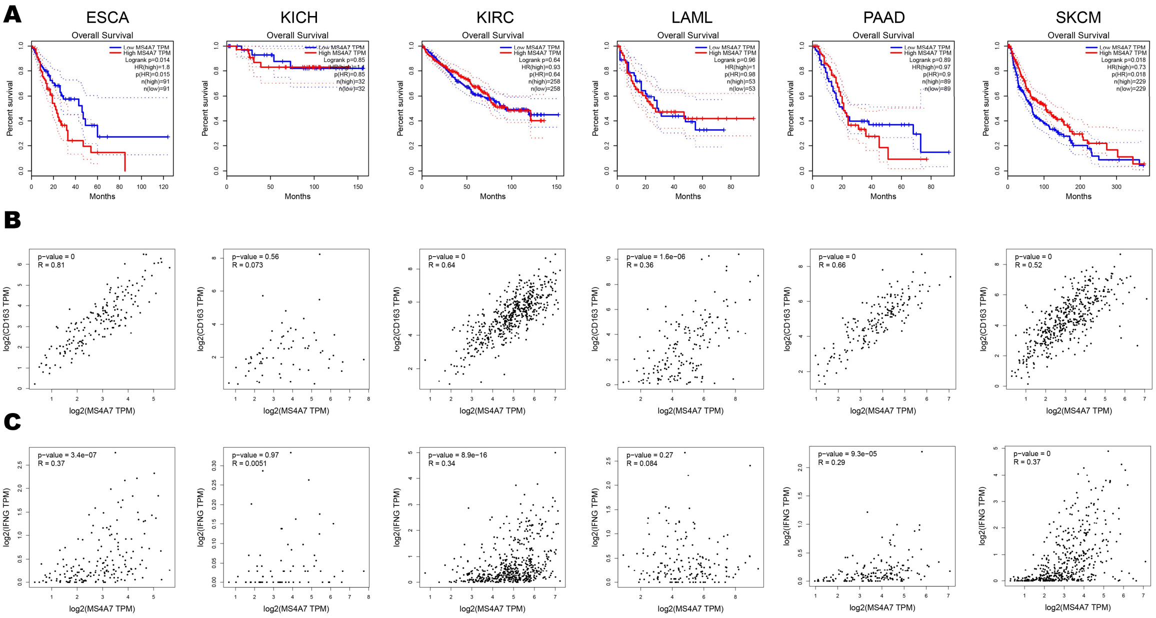

Figure S3

Pan-cancer analysis of MS4A7 based on TCGA data

(A) Kaplan–Meier survival curves stratified by the expression levels of MS4A7 based on TCGA ESCA, KICH, KIRC, LAML, PAAD and SKCM datasets. (B) The correlation of MS4A7 with CD163 mRNA expression was analyzed with Pearson's test. Data were obtained from the TCGA ESCA, KICH, KIRC, LAML, PAAD and SKCM datasets. (C) The correlation of MS4A7 with IFNG mRNA expression was analyzed with Pearson's test. Data were obtained from the TCGA ESCA, KICH, KIRC, LAML, PAAD and SKCM datasets.

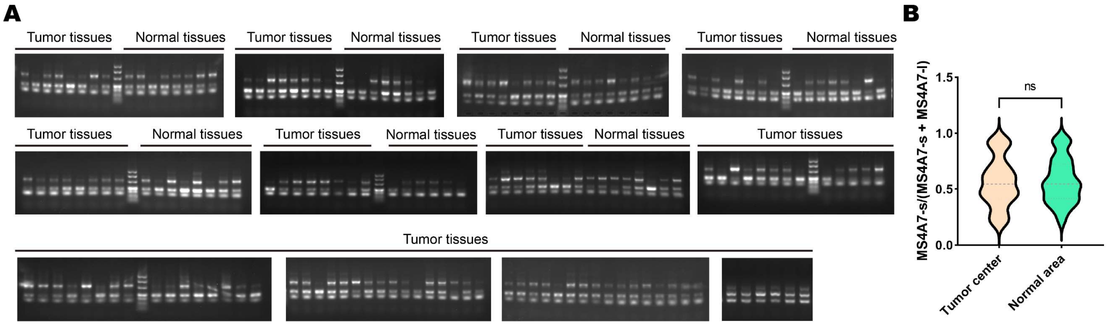

Figure S4

RT-PCR of MS4A7 isoforms mRNA levels in GBM (n = 123) and adjacent normal brain tissues (n = 54)

(A) RT-PCR of MS4A7 isoforms mRNA levels in GBM and adjacent normal brain tissues. (B) violin plot of MS4A7-s/ (MS4A7-s + MS4A7-l) ratio between GBM tissues and adjacent normal brain tissues. Statistical significances were analyzed by Student t test. ns: not significant.

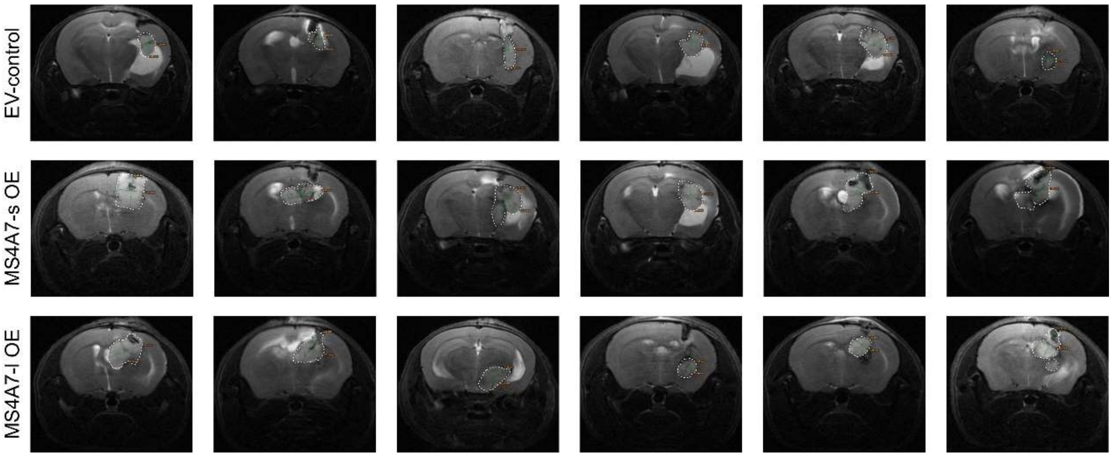

Figure S5

MRI images of tumors of EV-control group, MS4A7-s OE group, and MS4A7-l OE group

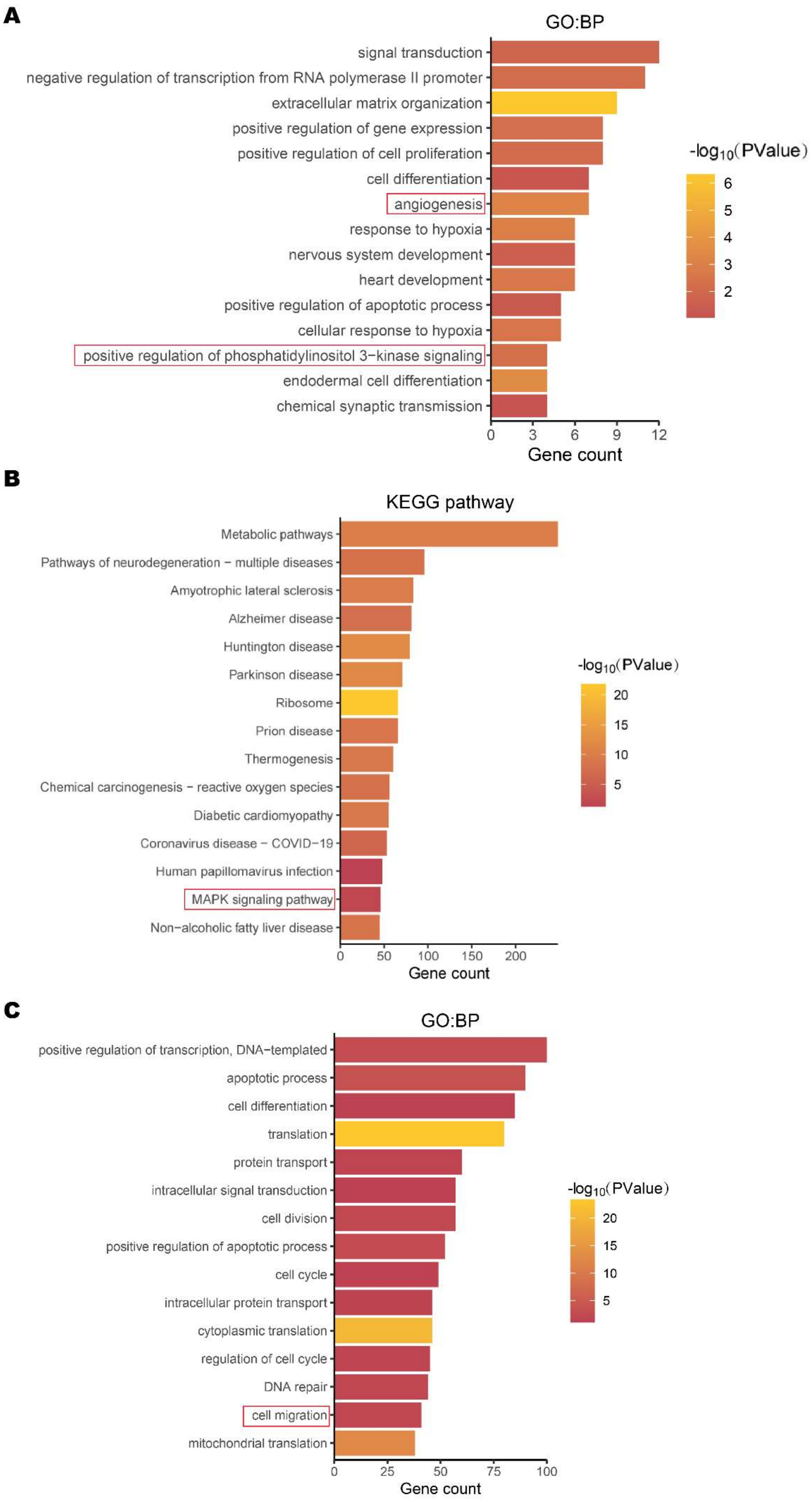
A
GO:BP
signal transduction
negative regulation of transcription from RNA polymerase II promoter
extracellular matrix organization
positive regulation of gene expression
positive regulation of cell proliferation
cell differentiation
angiogenesis
response to hypoxia
nervous system development
heart development
positive regulation of apoptotic process
cellular response to hypoxia
positive regulation of phosphatidylinositol 3-kinase signaling
endodermal cell differentiation
chemical synaptic transmission
0 3 6 9 12
Gene count
$-\log_{10}$(PValue)
6
5
4
3
2
B
KEGG pathway
Metabolic pathways
Pathways of neurodegeneration − multiple diseases
Amyotrophic lateral sclerosis
Alzheimer disease
Huntington disease
Parkinson disease
Ribosome
Prion disease
Thermogenesis
Chemical carcinogenesis − reactive oxygen species
Diabetic cardiomyopathy
Coronavirus disease − COVID-19
Human papillomavirus infection
MAPK signaling pathway
Non−alcoholic fatty liver disease
0 50 100 150 200
Gene count
$-\log_{10}$(PValue)
20
15
10
5
C
GO:BP
positive regulation of transcription, DNA−templated
apoptotic process
cell differentiation
translation
protein transport
intracellular signal transduction
cell division
positive regulation of apoptotic process
cell cycle
intracellular protein transport
cytoplasmic translation
regulation of cell cycle
DNA repair
cell migration
mitochondrial translation
0 25 50 75 100
Gene count
$-\log_{10}$(PValue)
20
15
10
5

Figure S6

Enrichment analyses of up-regulated genes of MS4A7-s OE and MS4A7-l OE group HMC3 cells

(A) Up-regulated pathway of MS4A7-s OE group based on GO:BP dataset. (B) Up-regulated pathway of MS4A7-l OE group based on KEGG dataset. (C) Up-regulated pathway of MS4A7-l OE group based on GO:BP dataset.
